# Cellular and population plasticity of helper CD4^+^ T cell responses

**DOI:** 10.3389/fphys.2013.00206

**Published:** 2013-08-16

**Authors:** Gesham Magombedze, Pradeep B. J. Reddy, Shigetoshi Eda, Vitaly V. Ganusov

**Affiliations:** ^1^National Institute for Mathematical and Biological Synthesis, University of TennesseeKnoxville, TN, USA; ^2^Department of Pathobiology, University of TennesseeKnoxville, TN, USA; ^3^Department of Forestry, Wildlife and Fisheries, Center for Wildlife Health, University of TennesseeKnoxville, TN, USA; ^4^Department of Microbiology, University of TennesseeKnoxville, TN, USA; ^5^Department of Mathematics, University of TennesseeKnoxville, TN, USA

**Keywords:** CD4^+^ T cells, differentiation, plasticity, mathematical modeling, Johnes disease

## Abstract

Vertebrates are constantly exposed to pathogens, and the adaptive immunity has most likely evolved to control and clear such infectious agents. CD4^+^ T cells are the major players in the adaptive immune response to pathogens. Following recognition of pathogen-derived antigens naïve CD4^+^ T cells differentiate into effectors which then control pathogen replication either directly by killing pathogen-infected cells or by assisting with generation of cytotoxic T lymphocytes (CTLs) or pathogen-specific antibodies. Pathogen-specific effector CD4^+^ T cells are highly heterogeneous in terms of cytokines they produce. Three major subtypes of effector CD4^+^ T cells have been identified: T-helper 1 (Th1) cells producing IFN-γ and TNF-α, Th2 cells producing IL-4 and IL-10, and Th17 cells producing IL-17. How this heterogeneity is maintained and what regulates changes in effector T cell composition during chronic infections remains poorly understood. In this review we discuss recent advances in our understanding of CD4^+^ T cell differentiation in response to microbial infections. We propose that a change in the phenotype of pathogen-specific effector CD4^+^ T cells during chronic infections, for example, from Th1 to Th2 response as observed in *Mycobactrium avium* ssp. *paratuberculosis* (MAP) infection of ruminants, can be achieved by conversion of T cells from one effector subset to another (cellular plasticity) or due to differences in kinetics (differentiation, proliferation, death) of different effector T cell subsets (population plasticity). We also shortly review mathematical models aimed at describing CD4^+^ T cell differentiation and outline areas for future experimental and theoretical research.

## Introduction

Adaptive immune responses are in general required for protection against many if not most pathogens. CD4^+^ T cells are the key component of adaptive responses to both intracellular and extracellular pathogens. The major function of CD4^+^ (helper) T cells is to provide help to other lymphocytes to mount an efficient immune response. By secreting appropriate cytokines and expressing a variety of co-stimulatory molecules, CD4^+^ T cells are required for the generation of high affinity antibody responses to pathogens and for the formation of long-lived plasma cells and memory B cells (Crotty, 2011). Although it is currently believed that CD4^+^ T cells are not needed for the generation of cytotoxic T lymphocyte (CTL) responses against many intracellular pathogens such as viruses (Wiesel and Oxenius, 2012), help from CD4^+^ T cells is required to generate memory CD8 T cells which are able to expand upon secondary exposure to the pathogen (Prlic et al., 2007). CD4^+^ T cells are in general needed to control chronic viral infections such as lymphocytic choriomeningitis virus (Zajac et al., 1998; Prlic et al., 2007; Zhang and Bevan, 2011). Recent evidence also suggests that CD4^+^ T cells could directly impact virus replication by killing virus-infected cells which express MHC-II molecules (Swain et al., 2012). By secreting a variety of cytokines, effector CD4^+^ T cells can also recruit other cells including neutrophils and monocytes to the sites of infection (Huber et al., 2012). CD4^+^ T cells are also involved in dampening immune responses either via the action of thymus-derived regulatory T cells (Tregs) or via production of anti-inflammatory cytokines such as IL-10 (Pot et al., 2011; Josefowicz et al., 2012).

How CD4^+^ T cells become activated, how they differentiate into effector cells, how effector phenotype of CD4^+^ T cells is maintained, and whether T cell effector phenotype can be changed to better control infections has been a subject of intensive research. In some circumstances, during progression of a chronic disease the efficient pathogen-specific CD4^+^ T cell response is lost and pathological response leading to exacerbation of the disease arises. Such a “switch” occurs during *Mycobactrium avium* ssp. *paratuberculosis* (MAP) infection of cattle and sheep where initially dominant MAP-specific cellular response (T-helper 1, Th1) is lost over time of infection, and MAP-specific antibody response (Th2) appears as the disease reaches clinical stage (Begg et al., 2011). In other circumstances, inappropriate responses arise following the first priming event. For example, exposure to allergens often leads to the generation of CD4^+^ T cell response that results in allergic reactions (Th2) rather than in protective immunity (Th1) (Holt and Thomas, 2005).

It is generally possible to bias differentiation on naïve CD4^+^ T cells into a particular effector T cell subset (e.g., Th1 or Th2) by providing appropriate environmental conditions. However, regulation of the phenotype of differentiated effector CD4^+^ T cells has proven to be more challenging. We propose that change of the phenotype of pathogen-specific CD4^+^ effector T cells during a chronic infection or a chronic inflammatory condition can be achieved via two distinct mechanisms: “cellular” and “population” plasticity of T cell effectors. We illustrate how mathematical modeling has been used to understand factors driving naïve CD4^+^ T cell differentiation and plasticity of effector T cell responses in chronic infections.

## Cellular and population plasticity of CD4^+^ T cell responses

### T cell differentiation

**Figure 1:**
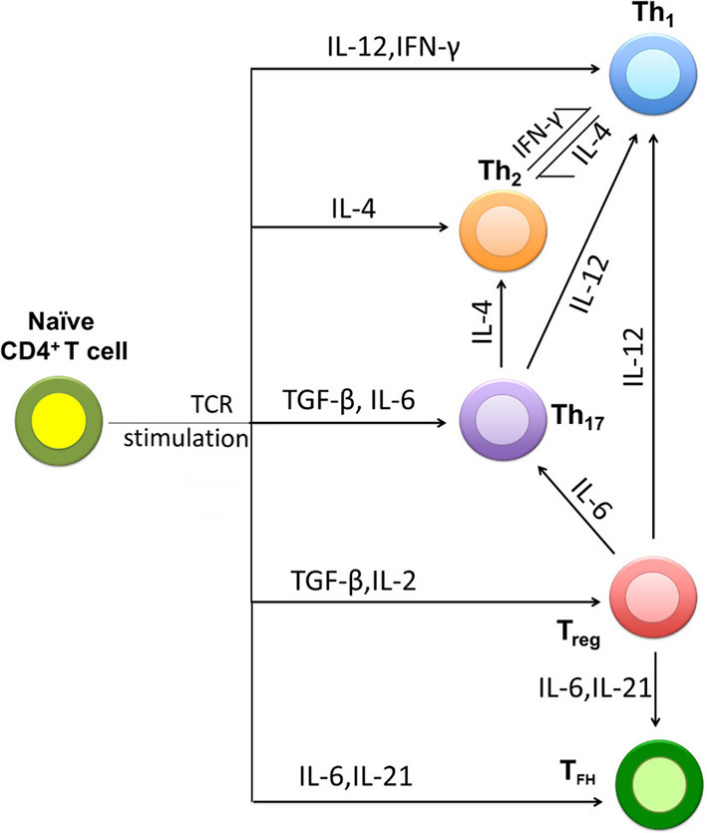
**Major pathways of naïve CD4^+^ T cell differentiation into effectors**. Upon encountering the antigens presented by the professional antigen-presenting cells (APCs) naïve CD4^+^ T cells differentiate into Th1, Th2, or Th17 effector cells. Cytokines present in the environment during differentiation play the major role in determining the phenotype that the CD4^+^ T cell will acquire. Two other CD4^+^ T cell subsets include regulatory T cells (Treg) and T follicular helper cells (Tfh). Due to cellular plasticity differentiated effector CD4^+^ T cells may convert from one type into another. For example, Th17 cells under strong polarizing conditions (e.g., high concentrations of IL-12) may convert into Th1 cells.

Naïve CD4^+^ T cells differentiate into various subsets upon interaction with an antigen presented by the professional antigen-presenting cells (APCs) such as dendritic cells (DC). CD4^+^ T cells require 3 signals for their lineage commitment (Kenneth et al., 2008). The first signal is generated following the interaction between T-cell receptor (TCR) and the peptide presented in the context of major histocompatibility complex (MHC) class II on an APC (Yamane and Paul, 2012). The second signal is generated following the interaction between the CD28 co-receptor on the T cell and B7 family of co-stimulatory molecules such as CD80 or CD86 on the APC. The third signal is generated by inflammatory cytokines produced by the APC or other cells at the site of T cell activation. These cytokines direct differentiation of naïve CD4^+^ T cells into a particular effector subset. Effector CD4^+^ T cells can be categorized into three major subsets based on the type of cytokine they produce and the major transcription factor (TF) they express (Figure 1). If an APC secretes interleukin (IL)-12, naïve CD4^+^ T cells differentiate into Th1 effectors. Th1 effectors express a transcription factor T-bet and secrete the cytokines IFN-γ and TNF-α; these cells play an essential role in inhibiting replication of intracellular pathogens such as viruses (Hsieh et al., 1993; Lighvani et al., 2001; Kenneth et al., 2008). If an APC secretes IL-4, naïve CD4^+^ T cells differentiate into Th2 effectors. Th2 cells express TF GATA-3, secrete cytokines IL-4, IL-5, and IL-13 (Le Gros et al., 1990; Eltholth et al., 2009); these cells are critical during infection by extracellular pathogens such as extracellular bacteria and helminthes. In the presence of IL-6 and transforming growth factor (TGF)-β, naïve CD4^+^ T cells differentiate into Th17 cells. Th17 cells express a transcription factor ROR-γt and produce cytokines IL-17 and IL-22 (Harrington et al., 2005; Ivanov et al., 2006); these cells are important for control of certain bacterial and fungal infections. Th1, Th2, and Th17 cells are considered to be the major effector CD4^+^ T cells (Mosmann et al., 1986; London et al., 1998; O'Garra, 1998; O'Garra and Arai, 2000; Yates et al., 2000; Murphy and Reiner, 2002; Chakir et al., 2003; Motiwala et al., 2006; Callard, 2007; Dong, 2008; Kenneth et al., 2008; Liao et al., 2011; Hong et al., 2012; Yamane and Paul, 2012).

Two other subsets of CD4^+^ T cells have been also identified (Figure 1). Tregs express TF FoxP3; these cells secrete anti-inflammatory cytokines like TGF-β and IL-10. Tregs maintain immune homeostasis by limiting the magnitude of immune response against pathogens and control inflammatory reactions (Sakaguchi, 2004). T follicular helper cells (Tfh) express a TF Bcl-6 and these cells are essential for the production of high affinity IgG antibodies (Crotty, 2011). Existence of Th9 and Th22 subsets was also recently suggested (Veldhoen et al., 2008; Eyerich et al., 2009).

### Cellular plasticity

It has been thought for a long time that differentiation of CD4^+^ T cells into various effector subsets is an irreversible event; CD4^+^ T cells that have differentiated into a particular subset cannot revert into a different subset (Mosmann and Coffman, 1989). However, recent studies suggest that effector T cells retain some degree of functional plasticity and these cells can change their effector phenotype (Murphy and Stockinger, 2010; O'Shea and Paul, 2010) (Figure 1). For example, recent reports have shown that both *in vitro* (Murphy et al., 1996) and *in vivo* (Panzer et al., 2012) generated Th1 cells can acquire the Th2 characteristics (Figure 1). Factors determining such *cellular plasticity* of CD4^+^ T cell effectors remain poorly understood. Experimental work suggests that plasticity of Th1 and Th2 subsets strongly depends on their differentiation state (Murphy et al., 1996) and that it is very difficult to reprogram the terminally differentiated subsets. For example, under some polarizing conditions Th2 cells cannot revert back to Th1 cells partly due to the loss of IL-12 receptor on these cells (Zhu and Paul, 2010). The definition “terminally differentiated CD4^+^ T cells” is very subjective, though. Long antigenic stimulation of naïve CD4^+^ T cells *in vitro* under either Th1 or Th2 polarizing conditions has been used as a surrogate for strong terminal differentiation. However, CD4^+^ T cells are rarely exposed to one polarizing cytokine environment *in vivo*. Recent work has also shown that Th1 cells are plastic; they can convert into Th2 cells in the presence of IL-4 (Szabo et al., 1995; Zhu and Paul, 2010). However, this conversion of the population of Th1 cells into Th2 effectors can also be explained by development of Th2 cells from naïve CD4^+^ T cells present in the Th1 cell population (Szabo et al., 1995). Recent studies also showed that the Th17 effector subset is unstable as compared to Th1 and Th2 effector cells, since Th17 cells can be reprogrammed to produce Th1 and Th2 cytokines (Lee et al., 2009). Furthermore, Tregs are plastic when cultured under Th1 (Oldenhove et al., 2009; Wei et al., 2009) or Th17 conditions (Yang et al., 2008a). Taken together, current data indicate that cellular plasticity of effector CD4^+^ T cell responses may be rather the rule than exception (Figure 1). How such plasticity is regulated remains poorly understood, however. Epigenetics is now considered to be one of the key mechanisms that dictates the stability and cellular plasticity of effector T cell subsets (Wilson et al., 2009).

Cellular plasticity of Th1 cells *in vivo* was demonstrated during *Nippostrongylus brasiliensis* infection during which the conversion of Th1 into Th2 cells was dependent on exogenous IL-4 (Panzer et al., 2012). Recent work suggests that conversion of Th1 into Th2 cells may occur independently of IL-4 via STAT-5-coupled cytokine receptors (Zhu et al., 2003, 2004). Furthermore, IL-4-independent conversion of Th1 into Th2 cells driven by signaling via the Notch receptor was also reported (Amsen et al., 2004, 2007).

Cell heterogeneity is a factor that can partially explain the plastic nature of effector CD4^+^ T cell subsets (Zhu and Paul, 2010). Such heterogeneity may arise when effectors can produce more than one cytokine. For example, while Th1 cells can produce IFN-γ, IL-2, and TNF-α, only a few of these cells express all the cytokines simultaneously (Darrah et al., 2007). Data from *in vitro* experiments (Murphy et al., 1996) showed that naïve CD4^+^ T cells differentiate into Th2 cells when stimulated with an antigen-loaded APCs in the presence of IL-4. However, even in such polarizing conditions a small percentage of cells in the cultures (4%) secrete IFN-γ. Similarly, in the presence of IL-12 and anti-IL-4 antibodies, only 80% of the cells were IFN-γ positive (Th1) and the rest, 20%, could either be undifferentiated or be cells producing IL-4 (Th2). Interestingly, using IL-4 to re-stimulate these strongly polarized Th1 cells induces IL-4 production in at least 8% of the population. The source of these IL-4 producing cells is unclear as they could have been derived from the undifferentiated naïve CD4^+^ T cells or from Th1 effectors. Taken together, recent work suggests that the phenotype of pathogen-specific effector CD4^+^ T cells may change over the course of infection due to cellular plasticity of T helper subsets. Yet, factors that regulate the efficiency at which the conversion from one cell subset to another occurs are still poorly understood.

### Population plasticity

**Figure 2:**
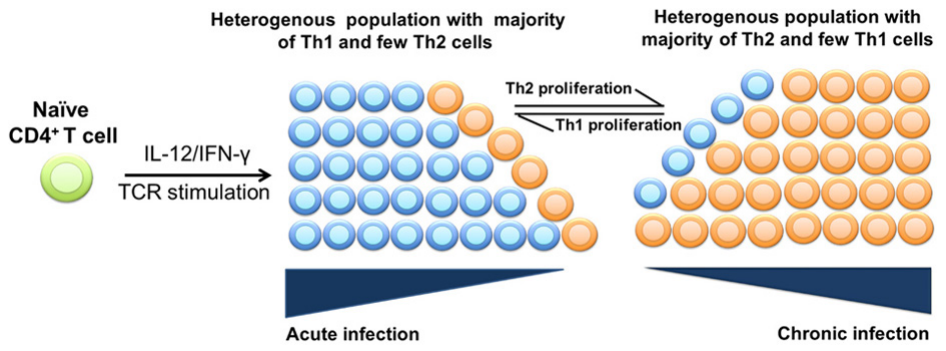
**Population plasticity of effector CD4^+^ T cells in chronic infections**. During an acute phase of infection, naïve CD4^+^ T cells differentiate into a heterogeneous population consisting mainly of Th1 cells and a few Th2 cells. However, as the disease progresses into a chronic phase, there is a gradual loss of Th1 cells and accumulation of Th2 cells. Accumulation of Th2 cells may occur due to a higher proliferation rate/reduced death rate of Th2 cells than that of Th1 cells.

Population plasticity is another major mechanism that may contribute to the change in the dominant phenotype of effector CD4^+^ T cells during chronic infections. In this mechanism, the size of the population of T cell effectors can increase due to preferential proliferation or reduced death of cells in the population (Figure 2). Generally, T cells undergo apoptosis under various conditions like cytokine deprivation (Cohen, 1993; Akbar et al., 1996), TNF-α level (Zheng et al., 1995), or a repeated stimulation with specific antigen due to activation-induced cell death (AICD) (Green and Scott, 1994; Kearney et al., 1994). Various reports claim the possibility of acquired tolerance with selective loss of Th1 cells and the persistence of Th2 cells (Burstein et al., 1992; De Wit et al., 1992). Additionally, the higher sensitivity of Th1 cells to AICD compared to Th2 counterparts was demonstrated (Ramsdell et al., 1994), which is likely to be removed due to a higher expression level of FasL in Th1 cells. The possibility of AICD of antigen-specific CD4^+^ T cell effectors during chronic infections was reported (Zhang et al., 1997). Once the majority of Th1 cells undergo apoptosis accompanied by the proliferation of Th2 cells (population plasticity), few Th1 cells that are present in the heterogeneous population could convert to Th2 subtype by epigenetic mechanisms (cellular plasticity). Population plasticity may be the major contributor to the change of the phenotype of the pathogen-specific T cells in chronic infections. Yet, the kinetics of proliferation and death of different subsets of effector CD4^+^ T cells during chronic infections are still lacking. Estimating the rates of proliferation, death, and re-differentiation of T effectors will lead to better quantitative understanding factors regulating the size of antigen-specific T cells in many pathological conditions.

### Th1/Th2 dynamics in chronic infections

**Figure 3:**
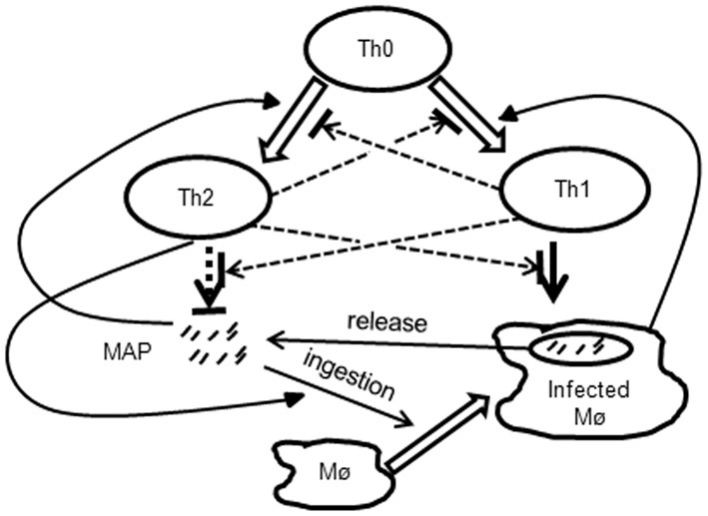
**Schematic representation of interactions between the bacteria and MAP-specific immune responses occurring JD**. During the infection, resting macrophages internalize extracellular MAP bacteria. Resting macrophages are unable to clear the bacteria, and after several rounds of replication macrophages rupture releasing more extracellular bacteria. Naïve CD4^+^ (Th0) cells differentiate either into Th1 or Th2 subsets depending on the density of infected macrophages or extracellular bacteria, respectively. Th1 and Th2 responses interact by inhibiting differentiation of naïve T cells and by reducing effector function of the opposite subset. Th1 response activates resting macrophages which are then able to clear the bacteria. Th2 response may contribute to the pathogenesis of the JD by increasing the uptake of extracellular bacteria by macrophages.

CD4^+^ T cell responses play a critical role in several chronic infections such as LCMV and HIV (Bevan, 2004; Wiesel and Oxenius, 2012; Streeck et al., 2013). The dynamics of pathogen-specific Th1 and Th2 responses has been studied during a mycobacterial infection with MAP called Johne's disease (JD, Figure 3). In early stages of MAP infection, Th1 cytokines such as IFN-γ, IL-2, and TNF-α, are highly expressed in serum of infected animals (Burrells et al., 1999; Stabel, 2000a), and culture of blood samples with MAP antigens lead to expansion of the population of IFN-γ producing CD4^+^ T cells. Expression of IFN-γ and TNF-α drives differentiation of naïve CD4^+^ T cells into Th1 effectors while suppressing differentiation of T cells into Th2 effectors (Harris et al., 2007; Amsen et al., 2009) (Figure 3). Th1 response via the production of IFN-γ plays a key role in controlling bacterial infection by promoting macrophage activation to kill intracellular bacteria and by up regulating MHC-II expression (Paludan, 1998). At later stages of MAP infection (clinical JD) infected animals shed a significant number of MAP in feces and produce a high level of anti-MAP serum antibody (Fecteau and Whitlock, 2010). Production of IFN-γ and IL-12 is generally reduced in cows with clinical JD (Stabel, 1996, 2000a; Burrells et al., 1999) whereas expression of a Th2 cytokine (IL-4) is elevated (Sweeney et al., 1998). IL-4 suppresses IFN-γ induced macrophage activation (Paludan, 1998) and inhibits autophagy-mediated killing of intracellular mycobacteria (Harris et al., 2007). These experimental findings suggest that during disease progression in MAP-infected animals there is a switch from the initially dominant MAP-specific cellular (Th1) response to the antibody (Th2) response (Stabel, 2000b).

What regulates the dynamics of this switch remains poorly understood, however. There are two possibilities: (1) the Th1/Th2 switch is the cause of disease progression and death of the infected animal, or (2) the Th1/Th2 switch is the consequence of disease progression which occurs independent of whether T-helper responses are present or not. How exactly Th1 response is lost and Th2 response arises is also unknown. In particular, the relative contribution of cellular vs. phenotypic plasticity of CD4^+^ T cell responses (Figures 1, 2) to the kinetics and likelihood of the Th1/Th2 switch in MAP-infected animals is not known. The issue is further complicated by the results of longitudinal studies on experimental infection of sheep with MAP that showed that the timing of Th1-Th2 switch varies between individual animals and that Th1 response [IFN-γ] may stay high even in late stages of MAP infection (Begg et al., 2011; Stabel and Robbe-Austerman, 2011).

The prevalence of apparently non-protective Th2 responses during a chronic infection occurs during leprosy caused by *Mycobacterium leprae* in humans. Similar to the MAP infection, leprosy is thought to be a dynamic process with changes in bacteria-specific cellular immune responses leading to clinical manifestations. *M. leprae* infects macrophages and their activation is a critical step for clearing the bacterial infection. When the infected macrophages are inactive, *M. leprae* evades the cellular immune response and replicates inside of the cell until the cell bursts. Without any external signal, macrophages are unable to mount any significant response to the bacteria, and the infection spreads largely unchecked. Macrophages are generally activated by IFN-γ-producing Th1 cells. Activated macrophages are more likely to kill intracellular bacteria by facilitating fusion of lysosomes with bacteria-harboring phagosomes (Kenneth et al., 2008). Patients with tuberculoid leprosy show very few lesions which are dominated by IFN-γ and very little bacteria can be recovered from the lesions. In the case of lepromatous leprosy, the infection is not contained, and there is a dominance of Th2 cell cytokines and elevated levels of anti-*M. leprae* antibodies in serum (Modlin, 1994). Reversal of cytokine pattern from Th2 to Th1 was reported during the shift from lepromatous leprosy to tuberculoid stage by administration of either IL-12 or IFN-γ to lepromatous patients (Modlin, 1994). Exact mechanisms by which such a therapy resulted in clearance of the pathogen from lesions remain poorly understood, but it may involve direct suppression of Th2 cell differentiation by IFN-γ, and therefore could arise due to population plasticity of CD4^+^ T cell responses (Modlin, 1994; Misra et al., 1995).

Modulation of the pathogen-specific effector T-helper responses has been also demonstrated in the case of Leishmaniasis, a disease caused by an infection with a protozoan *Leishmania major*. This parasite causes cutaneous leishmaniasis in mice and humans. Infection of mice with a low parasite dose leads to parasite containment associated with a Th1 type response, whereas infection with a high parasite dose leads to progressive disease associated with a Th2/antibody response (Menon and Bretscher, 1998). Similarly, humans with localized cutaneous leishmaniasis (LCL) display few lesions and the growth of the parasite is confined to the lesions. During diffuse cutaneous lesihmaniasis (DCL) the lesions are widely disseminated with an uncontrolled growth of the parasite. Th1 cytokines are dominant in LCL; they help in the elimination of the infection. However, in case of DCL prevalence of Th2 cytokines leads to uncontrolled growth of the parasite (Castellano et al., 2009). Whether the switch from the dominant Th2 response to the protective Th1 response in chronic infection is possible remains unclear, but it has been shown that clinical cure of patients with leishmaniasis occurs concomitantly with the loss of Th2 effectors and persistence of Th1 cells from the acute to the chronic stage of the disease (Castellano et al., 2009).

## Mathematical approaches in modeling CD4^+^ T cell differentiation

There have been many mathematical studies aimed at improving our understanding of mechanisms regulating T cell differentiation. Studies on mathematical modeling of Th1/Th2 responses can be categorized into three main subgroups.

The first subgroup of studies developed and analyzed mathematical models of differentiation of naïve CD4^+^ (Th0) cells into Th1 and Th2 subsets by including the dynamics of Th1/Th2 cytokines, intracellular molecules, and gene regulatory networks (Biedermann and Röcken, 1999; Fishman and Perelson, 1999; Yates et al., 2000, 2004; Bergmann et al., 2001, 2002; Richter et al., 2002; Bettelli et al., 2006; Callard, 2007; Fenton et al., 2008; Eftimie et al., 2010; Naldi et al., 2010; Vicente et al., 2010; Groß et al., 2011; Hong et al., 2011, 2012; Liao et al., 2011). Some of these models described differentiation of naïve CD4^+^ T cells into different effector T cell subsets via upregulation of the phenotype-specific TF (master regulators) such as T-bet, GATA-3, FoxP3, and ROR-γt (Höfer et al., 2002; Mariani et al., 2004; Yates et al., 2004; Callard, 2007; Van Den Ham and De Boer, 2008; Hong et al., 2011, 2012). These studies explained how positive and negative feedback loops between these master regulators result in differentiation of a particular subset of T effectors. Cytokines that are present in extracellular environment and are produced by effector T cells strongly influence the direction of naïve T cell differentiation. Signals provided by cytokines binding to cytokine receptors and by antigens binding to the T cell receptors are summarized internally and eventually determine the direction of cell differentiation. Some of the predictions of these mathematical models found confirmation in experimental papers (Zheng and Flavell, 1997; Chakir et al., 2003; Ivanov et al., 2006; Yang et al., 2008b; Liao et al., 2011; van den Ham et al., 2013). Further advances in understanding of T cell differentiation have been obtained using curated Boolean network models which included the dynamics of multiple genes in T cells such as those encoding for cytokines and cytokine receptors (Mendoza, 2006; Thakar et al., 2007; Kim et al., 2008; Santoni et al., 2008; Pedicini et al., 2010). Such multi-scale models capture communications between cells via cytokines and integrate intra- and extracellular dynamics of such signaling molecules (Santoni et al., 2008; Pedicini et al., 2010). Virtual deletion experiments of the key master regulators have been used to predict factors (e.g., TF, cytokines, or cytokine receptors) influencing differentiation of cells toward either Th1 or Th2 phenotype (Pedicini et al., 2010).

The second subgroup of studies modeled population plasticity of Th1/Th2 cell responses. These models included the processes of cross-regulation of Th1/Th2 cell responses either directly by cell-to-cell interactions or via production of Th1/Th2 cytokines (Fishman and Perelson, 1999; Yates et al., 2000, 2004; Bergmann et al., 2001, 2002; Fenton et al., 2008; Eftimie et al., 2010; Groß et al., 2011). Some of these models offered a theoretical explanation of the switch from an initially dominant pathogens-specific Th2 response to a later dominant Th1 response (*or vice versa*). These models, however, only focused on the dynamics of populations of CD4^+^ T cells and did not incorporate intracellular genetic and molecular networks that enable the cells to acquire different physiological states. For example, studies of Yates et al. (2000) and Bergmann et al. (2001) showed that when Th1 effectors fail to clear the antigen, initially dominant Th1 response is lost and Th2 response arises. In the Bergmann et al. (2001) model, the shift in dominance of effector T cell populations is regulated by differences in differentiation, cross-suppression and clonal expansion of each subset as the function of the antigen concentration. In the Yates et al. (2000) model, dominance of the particular effector T cell subset is driven by the level of Th1/Th2 cytokines. The latter model also investigated how population dynamics of T-helper responses is influenced by activation-induced cell death which limits clonal expansion and hence aids in resolving the T cell balance. It should be noted, however, that few if any of mathematical models in this subgroup have been developed to address the kinetics of effector T-helper responses during infections with biologically relevant pathogens.

The third subgroup of studies modeled cellular plasticity of effector CD4^+^ T cell responses. Mathematical models of this subgroup predict reversible phenotypic plasticity between effector Th17 cells to induced regulatory T cells (iTregs) and reprogramming of Th2-polarised cells to Th1 phenotype in Th1-polarising conditions (Naldi et al., 2010; Pedicini et al., 2010; Carbo et al., 2013). A typical example of such mathematical models is the work by Pedicini et al. (2010), which predicted master transcription regulators as attractors associated with development of Th1 and Th2 cells using a cytokine network model. This modeling study makes testable predictions on the mechanisms that regulate the balance between Th1 and Th2 cells and how loss of this balance can skew lineage selection. *In silico* virtual knockout experiments of GATA-3 predicted creation of attractors with high expression of IFN-γ. Furthermore, deletion of both T-bet and GATA-3 predicted increase in expression of several other non-specific Th2 TF such as IRF4, MAF, NFAT, STAT1, and STAT6. Although models in this subgroup often generate novel predictions these models are in general very complex involving description of tens of genes and their products. Predictions of these models will need to be tested in specifically designed experiments.

## Discussion

Discovery of several novel subsets of effector CD4^+^ T cells including Th17 and Tfh cells rejuvenated interest into factors that influence differentiation of naïve CD4^+^ T cells into effectors and the stability of different effector CD4^+^ T cell subsets both *in vitro* and following immune response to antigens *in vivo*. One of the most intriguing observations is that even differentiated effector CD4^+^ T cells can change their phenotype if the environmental conditions change (Murphy and Stockinger, 2010). Factors that regulate such *cellular plasticity* of effector and memory CD4^+^ T cell responses still remain incompletely defined, and how and whether such plasticity can be explored therapeutically is unknown.

In a number of conditions including infections, autoimmune diseases, and allergic reaction, the host generates an effector CD4^+^ T cell response of inadequate phenotype that may lead to worsening of symptoms and often to exacerbation of the disease. In particular, during MAP infection of ruminants initially protective Th1 CD4^+^ T cell response is lost over time, and non-protective Th2 response arises (Stabel, 1996, 2000a; Burrells et al., 1999). What regulates this change in the immune response phenotype is unclear. Conversion of MAP-specific Th1 cells into Th2 over time (cellular plasticity) could be one potential mechanism. Alternatively, there may be quantitative differences in the rates of differentiation of naïve CD4^+^ T cells into two subsets of effectors, differences in the rates of proliferation, death, and migration of different subsets of CD4^+^ T cells to the site of infection (population plasticity). Finally, phenotype switch could be driven by other helper cell types, for example, thymus-derived Tregs or periphery-induced Tregs. Experimentally, it will be a challenge to discriminate between these alternative mechanisms of Th1/Th2 switch during JD. As for other conditions (e.g., allergic reactions) mechanisms driving the change in phenotype of allergen-specific CD4^+^ T cell effectors following immunotherapy remain to be determined (Holt and Thomas, 2005; van Oosterhout and Motta, 2005). We believe that one of the important experimental challenges is to evaluate the rates at which different effector T-helper cell subsets proliferate and die during chronic inflammatory conditions (e.g., infections) and whether these rates are influenced by the type of inflammatory environment.

Many mathematical models on CD4^+^ T cell differentiation have been developed and analyzed. The vast majority of these models focus on the initial differentiation step of naïve CD4^+^ T cells into a particular effector subset. Such models are useful for the vaccine development where induction of an appropriate CD4^+^ T cell response will be critical for the vaccine efficacy. The discovery of cellular plasticity of effector CD4^+^ T cells calls for the need to develop novel mathematical models that explain and predict how one T cell subset is converted into another subset. The use of gene expression and phenotypic data from *in vitro* and *in vivo* generated effector CD4^+^ T cells will be instrumental for testing and verifying such mathematical models.

Mathematical models have also been developed to explain population plasticity of effector T cell responses. These models are more relevant to chronic conditions such as persistent infections and autoimmune diseases. Yet, most of these models have been poorly parameterized and predictions of such models have not been adequately tested in well-designed experiments. More experimental data is needed to explain how proliferation, death, and differentiation of effector T cells are influenced by the environment and the subsets themselves. Also, data on the dynamics of effector T cells at the sites of infection will be useful for the development of models for specific infections. In all cases, development of quantitative mathematical models can be greatly enhanced by closer collaborations between mathematicians/modelers and wet-lab experimentalists.

### Conflict of interest statement

The authors declare that the research was conducted in the absence of any commercial or financial relationships that could be construed as a potential conflict of interest.
